# Long-Term Conditioning to Elevated pCO_2_ and Warming Influences the Fatty and Amino Acid Composition of the Diatom *Cylindrotheca fusiformis*

**DOI:** 10.1371/journal.pone.0123945

**Published:** 2015-05-13

**Authors:** Rafael Bermúdez, Yuanyuan Feng, Michael Y. Roleda, Avery O. Tatters, David A. Hutchins, Thomas Larsen, Philip W. Boyd, Catriona L. Hurd, Ulf Riebesell, Monika Winder

**Affiliations:** 1 GEOMAR | Helmholtz Centre for Ocean Research Kiel, Kiel, Germany; 2 Facultad de Ingeniería Marítima, Ciencias Biológicas, Oceánicas y Recursos Naturales, Escuela Superior Politécnica del Litoral, Guayaquil, Ecuador; 3 Department of Botany, University of Otago, Dunedin, New Zealand; 4 Bioforsk Norwegian Institute for Agricultural and Environmental Research, Bodø, Norway; 5 Marine and Environmental Biology, University of Southern California, Los Angeles, California, United States of America; 6 Leibniz-Laboratory for Radiometric Dating and Stable Isotope Research, Christian-Albrechts-Universität zu Kiel, Kiel, Germany; 7 Institute for Marine and Antarctic Studies, University of Tasmania, Hobart, Australia; 8 Department of Ecology, Environment and Plant Sciences, Stockholm University, Stockholm, Sweden; Stazione Zoologica Anton Dohrn, Naples, ITALY

## Abstract

The unabated rise in anthropogenic CO₂ emissions is predicted to strongly influence the ocean’s environment, increasing the mean sea-surface temperature by 4°C and causing a pH decline of 0.3 units by the year 2100. These changes are likely to affect the nutritional value of marine food sources since temperature and CO₂ can influence the fatty (FA) and amino acid (AA) composition of marine primary producers. Here, essential amino (EA) and polyunsaturated fatty (PUFA) acids are of particular importance due to their nutritional value to higher trophic levels. In order to determine the interactive effects of CO₂ and temperature on the nutritional quality of a primary producer, we analyzed the relative PUFA and EA composition of the diatom *Cylindrotheca fusiformis* cultured under a factorial matrix of 2 temperatures (14 and 19°C) and 3 partial pressures of CO₂ (180, 380, 750 μatm) for >250 generations. Our results show a decay of ~3% and ~6% in PUFA and EA content in algae kept at a pCO₂ of 750 μatm (high) compared to the 380 μatm (intermediate) CO₂ treatments at 14°C. Cultures kept at 19°C displayed a ~3% lower PUFA content under high compared to intermediate pCO₂, while EA did not show differences between treatments. Algae grown at a pCO₂ of 180 μatm (low) had a lower PUFA and AA content in relation to those at intermediate and high CO₂ levels at 14°C, but there were no differences in EA at 19°C for any CO₂ treatment. This study is the first to report adverse effects of warming and acidification on the EA of a primary producer, and corroborates previous observations of negative effects of these stressors on PUFA. Considering that only ~20% of essential biomolecules such as PUFA (and possibly EA) are incorporated into new biomass at the next trophic level, the potential impacts of adverse effects of ocean warming and acidification at the base of the food web may be amplified towards higher trophic levels, which rely on them as source of essential biomolecules.

## Introduction

Anthropogenic activities during the past 250 years have almost doubled the atmospheric CO_2_ concentration and strongly influenced the ocean’s physical and chemical environment. It has been projected that by the year 2100, the mean sea surface temperature will increase by 1–4°C and pH decrease by 0.3 units through alterations of the carbon chemistry of seawater [1]. Ocean warming and acidification are affecting a wide range of marine organisms in various ways [2,3]. They can also affect the macromolecular composition of primary producers [4,5] and consequently the nutritional value for higher trophic levels that depend upon them as a source of essential biomolecules [6–8].

Lipids and proteins have crucial structural and physiological roles in all living organisms and both are composed of subunits known as fatty (FA) and amino acids (AA). FA consist of hydrocarbon chains of different lengths and saturation (number of double bonds); they are generally classified in saturated (SFA, no double bonds), monounsaturated (MUFA, one double bond) and polyunsaturated (PUFA, with two or more double bonds) [9]. AA are composed of a carbon backbone with a side-chain specific to each AA containing one or two amino groups [10]. From a nutritional and physiological perspective, amino acids can be characterized as non-essential (NEA) and essential (EA) compounds [11]. The PUFA and EA have a particular ecological relevance as they cannot be synthesized *de novo* by metazoans, and therefore have to be acquired from dietary sources [12,13].

The macromolecular composition of individual algal species is influenced by environmental variables such as nutrient availability [14–16], temperature [4,5,16], and CO_2_ concentration [5,17]. The FA composition of marine algae, especially PUFA, can be affected by temperature and CO_2_. For example, marine algae regulate their FA composition and the degree of desaturation in response to changing temperature in order to keep a steady membrane fluidity [9], with the amount of PUFA being generally inversely proportional to temperature [18]. In contrast, the effects of high CO_2_ on algal FA content, particularly PUFA, seem to be more diverse and species-specific, ranging from declining to increasing PUFA concentration [5,17,19,20]. The mechanisms through which CO_2_ affects algal FA are unclear, however it has been suggested that high CO_2_ levels enhance SFA synthesis and accumulation [21], which reduces cell membrane fluidity in order to cope with changing ambient pH conditions and facilitates the regulation of cell homeostasis [7,22].

There is little information regarding the effects of temperature and CO_2_ on algal AA composition. Higher temperatures can increase the protein content in algae [4,23], and EA contents show an optimum curve, with cellular EA increasing with temperature up to a point and decreasing thereafter [24]. Some studies have shown that CO_2_ can affect the protein content of marine algae [23] and that EA seems to be more abundant at low CO_2_ conditions [25]. This CO_2_-induced change in EA has been attributed to reduced amounts of protein content, for instance those proteins related to active CO_2_ uptake such as the mitochondrial carbonic anhydrase in the algae *Chlamydomonas reinhardtii* [25]. However the above mentioned experiments were conducted using extremely high CO_2_ levels (5000 ppm) that are unrealistic in natural environments and that far exceed forecasted future oceanic pCO_2_ levels [1]. Therefore, these results cannot be easily extrapolated to understand AA metabolism in future ocean acidification scenarios.

Temperature and CO_2_-driven changes in algal FA and AA composition are ecologically relevant, as qualitative traits in the food source are often more important than their quantity in trophic transfer efficiency and production at higher trophic levels [26,27]. Several laboratory and field studies have shown that a decline of PUFA and/or AA in marine primary producers can directly affect intermediate level grazers in food webs. For instance, for copepods deficiencies in these essential biomolecules can have serious consequences for development and egg production rates [7,12,28,29]. Furthermore, it has been show that the FA and AA composition of primary producers and secondary consumers are transferred and amplified to higher trophic levels of the food web. In this way biomolecular differences can affect the physiology of larval fish, which rely heavily on the quality of their food source for essential macromolecules [30,31].

In spite of the importance of marine food webs to provide essential resources for human consumption [32], the understanding of the influence of ocean warming and acidification on primary producers in terms of their quality as a source of essential macromolecules is limited. To fill this knowledge gap, we investigated the interactive effects of CO_2_ and temperature on the relative FA and AA composition in marine algae, with emphasis on PUFA and EA. A freshly isolated strain of the diatom C*ylindrotheca fusiformis* c.f. [33] was kept in a factorial design at three CO_2_ levels and two temperature regimes for >250 generations, and their FA and AA composition measured. A diatom was chosen for this study as this group is one the most abundant taxa in marine phytoplankton and forms the base of some of the most productive ecosystems in the world.

## Materials and Methods

### Algal material

*Cylindrotheca fusiformis* Reimann and Lewin 1964 was isolated from water collected approximately 3 km offshore from Tairoa Head at the mouth of Otago Harbor halfway along the ‘Munida transect’ (45° 45’ 09”S and 170° 48’ 6”E) off Dunedin, New Zealand [34] in January of 2011. The ambient sea surface temperature was 14.8°C. The culture was established as described in [33]. Two to four cells were originally isolated from each treatment combinations (pCO_2_ and temperature) and propagated separately in wells of a 24-well plate. Only one strain was used for the long-term maintenance under pCO_2_ and temperature conditions identical to those from which they were isolated.

### Experimental set up

The culture was maintained for 1 year in full factorial design consisting of three CO_2_ concentrations (180, 380 and 750 μatm pCO_2_) and two temperatures (14 and 19°C). The CO_2_ concentrations correspond to the pre-industrial (180), current (380) and that projected for the year 2050 (750), respectively[1]. 14°C is the average surface seawater temperature in summer at the collection site of *C*. *fusiformis* [33], the +5°C manipulation was selected based on projected sea surface warming according to Rhein et al. [1]. Each of the 6 treatment combinations was prepared in triplicate. The cultures were incubated in eighteen 500-mL polycarbonate bottles and were gently bubbled at each temperature using corresponding commercially prepared air/CO_2_ mixtures (Alphagaz, Air Liquide) following published protocols [35,36]. Cultures were grown under a 12h:12h L:D photoperiod at 120 μmol photons m^-2^ s^-1^ photosynthetically active radiation with cool white fluorescence lamps as a light source.

The algae were kept in active growth using semi-continuous culture methods for over 250 generations before sampling. The culture media was prepared from 0.2μm filtered seawater enriched with f/50 nutrient according to [37] consisting of 10 μmol L^-1^ NaNO_3_^-^, 0.8 μmol L^-1^ NaH_2_PO_4_^3-^, 10 μmol L^-1^ Na_2_SiO_3_ with f/50 vitamin and trace metal concentrations (25 times dilution of f/2 recipe). The cultures in each incubation bottle were diluted constantly every three days during the course of the semi-continuous incubation with freshly made seawater medium. The seawater medium for dilution was pre-bubbled using the commercially prepared air/CO_2_ mixtures at the appropriate *p*CO_2_ for each experimental treatment. The cell abundance was kept at <10^4^ cells mL^-1^ throughout the incubation, in order to minimize biological effects on seawater carbonate chemistry. At the end of the experimental period, 100 ml subsample for FA and AA analysis from each replicate was filtered onto a polycarbonate filter and stored at -80°C until analysis.

During the long-term maintenance of the culture, the doubling time and growth rate were established early on under the different treatment combinations; thereafter the culture was grown semi-continuously with appropriate dilution factor to obtain the target range of cell density. Samples were obtained several times before and after dilution for immediate counting and verification. Upon termination of the experiment, algal samples for cell counts were collected in 30 ml borosilicate glass scintillation vials, preserved with acidified Lugol’s solution and enumerated with a phytoplankton counting chamber (PhycoTech, USA) using a Zeiss microscope (Axiostar plus, Germany) at magnification of ×200. Cell-specific growth rates for each treatment level were calculated as μ = ln(N_1_-N_o_)/(t_1_-t_0_), where N_1_ and N_0_ represent the cell abundances at time t_1_ and t_0_ (in days).

### Seawater carbonate system

Samples for the analysis of the carbonate system parameters were taken at the end of the experiment. pH was measured spectrophotometrically as described in McGraw et al. [38] using a UV–vis spectrophotometer (Ocean Optics USB4000). Total alkalinity was measured at 25°C, applying a two-stage potentiometric open cell titration [39] and corrected with certified reference material (A. Dickson, La Jolla, California). Temperature was monitored using a standard laboratory incubator thermometers and salinity by conductivity with an interchangeable probe using an Orion 5-star plus pH meter. Experimental pCO_2_ was calculated using pH and total alkalinity in the CO2SYS software [40] with dissociation constants described by Tatters et al. [33].

### Fatty acid quantification

FA were measured by gas chromatography (GC) as fatty acid methyl esters (FAME) following [41]. Lipids were extracted overnight from the filters using 3ml of a solvent mixture (dichloromethane:methanol:chloroform in 1:1:1 volume ratios). As an internal standard, FAME C19:0 (Restek, Bad Homburg, Germany; c = 20.0 ng component^-1^μl^-1^) was added, and a C23:0 FA standard (c = 25.1 ng μl^-1^) used as an esterification efficiency control (usually 80–85%). Water-soluble fractions were removed by washing with 2.25 ml of KCl solution (c = 1 mol L^-1^), and the remainder dried by addition of NaSO_4_. The solvent was evaporated to dryness in a rotary film evaporator (100–150 mbar), re-dissolved in Chloroform and transferred into a glass cocoon. The solvent was evaporated again (10–30 mbar), and esterification was performed overnight using 200 μl 1% H_2_SO_4_ (in CH_3_OH) and 100 μl toluene at 50°C. Phases were split using 300 μl 5% sodium chloride solution, and FAMEs were separated using n-Hexane, transferred into a new cocoon, evaporated, and 100 μl (final volume) added. All solvents used were gas chromatography grade. FAMEs were analyzed by a Thermo GC Ultra gas chromatograph equipped with a non-polar column (Restek RXI1-SIL-MS 0.32 μm, 30 m) using a Flame ionization detector. The column oven was initially set to 100°C, and heated to 220°C at 2°C min^-1^. The carrier gas was helium at a constant flow of 2 ml min^-1^. The flame ionization detector was set to 280°C, with a gas flow of 350, 35 and 30 ml min^-1^ of synthetic air, hydrogen and helium, respectively. A 1 μl aliquot of the sample was injected. The system was calibrated with a 37-component FAME-mix (Supelco, Germany) and chromatograms were analyzed using Chrom-Card Trace-Focus GC software [41] and the fatty acids were clustered according to their degree of saturation: saturated (SFA), monounsaturated (MUFA) and polyunsaturated (PUFA) and calculated as the relative content (%) of each group in relation to the others.

### Amino acid quantification

Amino acids were quantified with the PhenomenexEZ:faast kit for a GC flame ionization detector (Torrance, CA, USA) following the protocol described in [42]. The samples were first thoroughly washed off the polycarbonate filters into Pyrex culture tubes (13 × 100 mm), hydrolyzed with 1 ml 6 N HCl (37% HCl analytical grade from Merck, Darmstadt diluted with purified water) flushed with N_2_, sealed with PTFE lined heat resistant caps, and placed in a heating block at 150°C for 70 minutes. After hydrolysis, the samples were dried in a heating block at 110°C for 30 minutes under a gentle stream of N_2_. Before the derivatization, 200 μM norvaline in an acidified solution were added as an internal standard, and the samples were then cleaned with a proprietary cation-exchange mechanism (solid-phase extraction) from Phenomenex. The samples were then washed with 1-propanol and H_2_O, eluted with a solution of aqueous NaOH, 1-propanol and 3-picoline, derived with a solution of CHCl_3_, 2,2,4-trimethylpentane and propylchloroformate. In the final step, the derivative amino acids were diluted with a HCl solution [42]. The final solution was injected into an Agilent 6890N GC with a 10 m x 0.25mm Zebron EZ-AAA column and a flame ionization detector. The GC settings were as follows: 2 μl of each sample was injected with a 1:15 ratio into a 250°C liner, the carrier gas was set to constant flow at 1.5 ml H_2_ min^-1^ and the oven program was 32°C min^-1^ from 110°C to 320°C. A standard solution with 23 amino acids (Phenomenex) was used as an internal reference. Of the amino acids we were able to analyze the following were defined as non-essential (NEA): alanine (Ala), asparagine/aspartic acid (Asx), glutamine/glutamic acid (Glx), glycine (Gly), and tyrosine (Tyr). The following were defined as essential (EA) for heterotroph consumers: histidine (His), isoleucine (Ile), leucine (Leu), lysine (Lys), methionine (Met), phenylalanine (Phe), threonine (Thr), and valine (Val). Their concentration was calculated as the relative content (%) of each group in relation to the other.

### Statistical analysis

A two-way ANOVA was used to test for differences between experimental treatments on the response variables AA and PUFA: differences were considered significant at p<0.05. Homogeneity of variance was check with a Bartlettor Fligner test, normality of distribution with a Shapiro test, and post-hoc analysis was performed with a Tukey test. A principal component analysis (PCA) was used to determine the difference in individual FA and AA composition of algae across the treatment combinations. To reduce unexplained variance in the PCA, a threshold of >1% in relative concentration of the individual FA and AA was set for its inclusion in the analysis. All statistical analyses were done using the R software environment 3.0.1 [43].

## Results

The pH in the algal cultures was within the expected pH range (S1A Fig) and although the calculated pCO_2_ at the end of the experiments was lower than the target values, it was consistently different between the treatments (S1B Fig). There was no significant effect of CO_2_ or temperature on diatom growth rate (S1C Fig).

### Fatty acids

**Fig 1:**
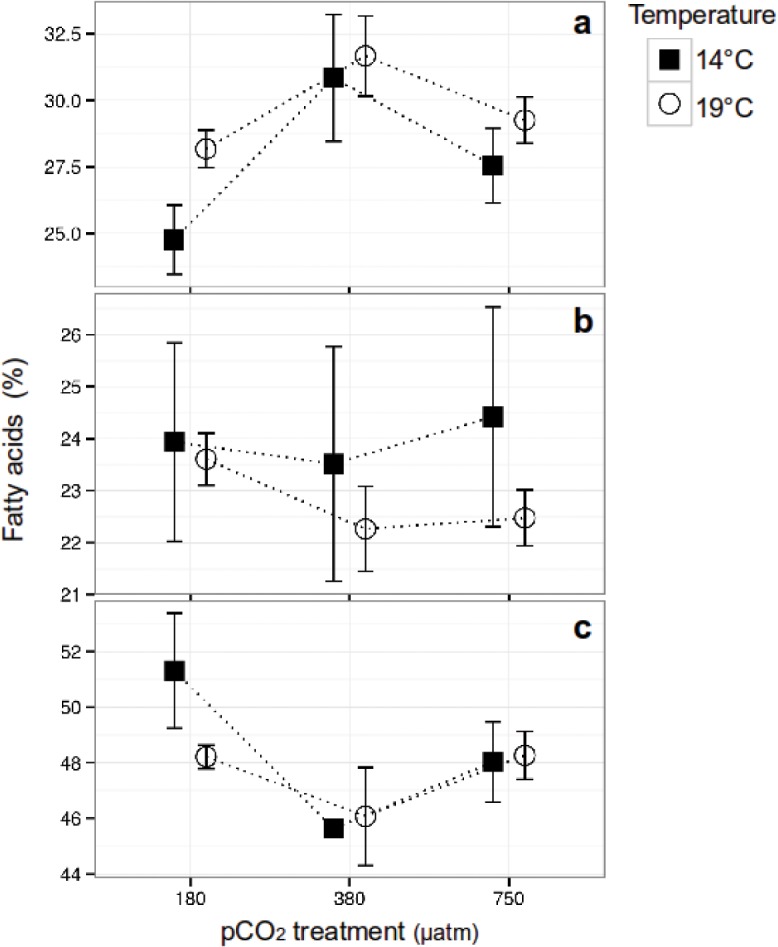
Relative fatty acid content in *Cylindrotheca fusiformis*. (a) Polyunsaturated [PUFA], (b) Monounsaturated [MUFA] and (c) Saturated [SFA] fatty acids, cultured under three CO_2_ levels and two temperatures conditions for >250 generations. a) PUFA showed a significant CO_2_ and temperature effect with a maximum at the intermediate CO_2_ treatment and an overall higher concentrations at 19°C. b) MUFA showed no differences between the treatments. c) SFA showed a significant difference related to CO_2_ but no temperature effect. Error bars denote ± 1 standard deviation (n = 3).

A total of 24 individual FA were identified and measured in the diatom *C*. *fusiformis* (S1 Table). The FA profile consisted of ~27% PUFA, ~ 23% MUFA and ~50% SFA across the treatments (Fig 1). The analysis of the FA groups showed no significant interactive effects of pCO_2_ and temperature on the relative concentration of any of the three groups after 1 year of acclimation. The relative PUFA content was significantly affected by CO_2_ (two-way ANOVA, F = 16.32, p<0.001, df = 2) with a maximum proportion at 380 in relation to the 180 (~ +5%) and 750 μatm CO_2_ (~ +3%) treatments (post-hoc 180–380: p<0.001; 380–750: p<0.05). Temperature also showed a significant effect (two-way ANOVA, F = 8.3, p<0.05, df = 1) primarily driven by higher PUFA relative content at 19°C compared to 14°C (~+3%) (post-hoc: p<0.05) under 180 μatm CO_2_ (Fig 1A). MUFA did not show significant differences between the treatments (Fig 1B). The SFA relative content differed between CO_2_ treatments (two-way ANOVA, F = 13.24, p<0.001, df = 2) with the lowest concentration at 380 in relation to the 180 (~ -6%) and 750 μatm CO_2_ (~ -2%) treatments (post-hoc 180–380: p<0.001; 380–750: p<0.05), although there were no significant temperature effects (Fig 1C). The most abundant PUFA was Eicosapentaenoic acid (EPA, 20:5n3c) (S1 Table), which amounted almost to half of the total PUFA in the samples and was strongly influenced by CO_2_ (S2A Fig).

**Fig 2:**
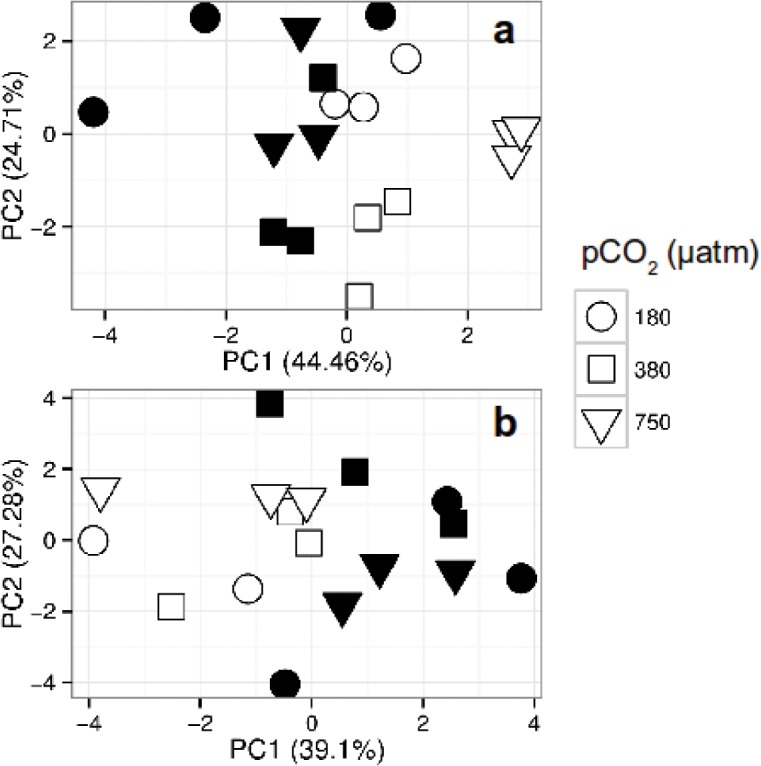
Principal component analysis [PCA] of the fatty acids [FA] and amino acids [AA] in *Cylindrotheca fusiformis*. The algae was cultured under three different CO_2_ conditions and two temperatures (19°C in black and 14°C in white) for >250 generations. Only the FA and AA with a concentration above 1% were included in the analysis. a) The abundance of essential polyunsaturated fatty acids [PUFA] was higher at 19°C (PC1) and intermediate CO_2_ level (PC2), however the influence of the former was lower. b) A higher abundance of essential amino acids [EA] was more common at high temperature (PC1) and intermediate CO_2_ level (PC2), although the CO_2_ influence was comparatively smaller.

The PCA of the individual algal FA compounds showed that PUFA had higher similarity in terms of relative abundance at high temperatures (PC1, 44.4%) and intermediate CO_2_ level (PC2, 24.7%), however the influence of the former was lower (Fig 2A). The axis loads of the PCA analysis showed that the PUFA had a strong influence on the explained variance of both axes (S3A Fig).

### Amino acids

**Fig 3:**
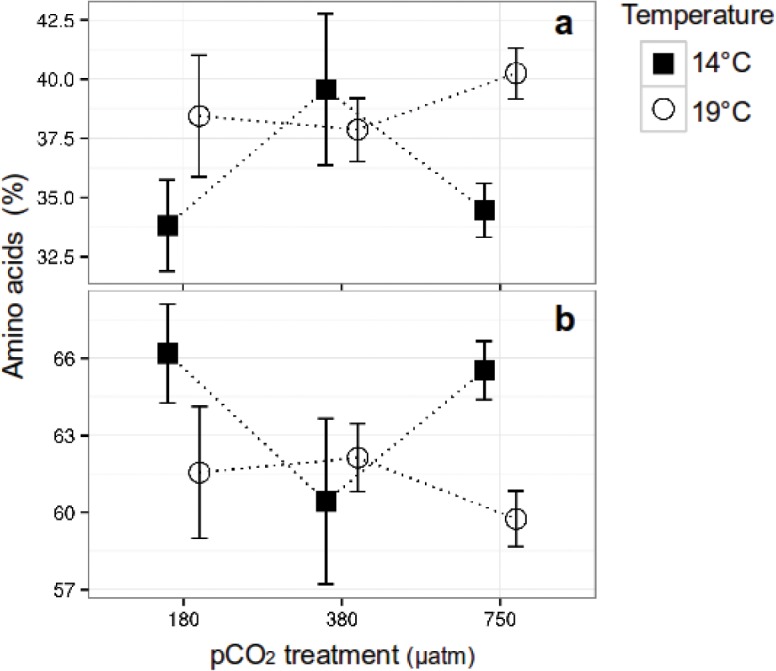
Relative amino acid content in *Cylindrotheca fusiformis*. (a) Essential [EA] and (b) Non-essential [NEA] amino acids, cultured under three CO_2_ conditions and two temperatures for >250 generations. a) EA showed a significant interaction between CO_2_ and temperature. Additionally, temperature alone showed a significant difference, and although CO_2_ affected its concentration, it was not significant. b) NEA showed the same effects as observed in EA. Error bars denote ± 1 standard deviation (n = 3, with exception of the 180 CO_2_-19°C treatment, where n = 2).

A total of 12 AA were identified and quantified (S2 Table) in *C*. *fusiformis*. The analysis of the specific AA groups showed that EA represented ~40% and NEA the remaining ~60%. After 1 year of acclimation, CO_2_ and temperature interactively affected the relative concentration of both AA groups (two-way ANOVA, F = 6.1, p<0.05, df = 2, for both) (Fig 3A and 3B).

Temperature influenced the relative content of both the EA and NEA groups (two-way ANOVA, F = 8.3, p<0.05, df = 1, for both groups), and CO_2_ affected their concentration but was statistically non-significant (two-way ANOVA, F = 3.2, p = 0.07, df = 2, for both). At 380 μatm CO_2_, EA showed their maximum relative concentration at 14°C compared to the 180 (~ +6%) and 750 μatm CO_2_ (~ +5%) treatments (Fig 3A), however at 19°C their concentration was significantly higher at 750 μatm CO_2_ (~ +6%) (post-hoc p<0.05), while the NEA followed the opposite trend (Fig 3B). Leucine (Leu) was the most abundant EA and represented ~10% of the total AA and ~25% of the total EA (S2B Fig). A post-hoc analysis of all treatment combinations is given in S3 Table.

The PCA of the AA composition of the diatom showed a higher similarity in terms of relative abundance of EA at high temperature (PC1, 39%) and intermediate CO_2_ level, although its influence was comparatively smaller (PC2, 27%) (Fig 2B). The axis loads showed that EA abundance strongly drove the differences in the AA profile composition of the treatments on both axes (S3B Fig).

## Discussion and Conclusions

### Fatty acids

Our observations of the *C*. *fusiformis* FA composition are comparable to results reported in other diatoms kept under diverse pCO_2_ and temperature conditions. The PUFA group showed a maximum in relative abundance at current environmental CO_2_ conditions, decreasing at values above and below present values, independently of the temperature treatment. This indicates that CO_2_ had a stronger influence than temperature on the relative algal PUFA content. A recent study showed that elevated CO_2_ significantly changed the FA concentration and composition of the diatom *Thalassiosira pseudonana*, causing a significant PUFA decline of ~20% in the algae cultured under elevated (750 μatm) compared to present day (380 μatm) CO_2_ levels during a short-term experiment [7]. Likewise, an interactive effect of temperature and CO_2_ was reported on the Antarctic sea ice diatom *Nitzschia lecointei* where cellular PUFA content was reduced by 40% at 960 μatm pCO_2_ relative to 390 μatm pCO_2_ when cultured at −1.8°C, and the PUFA content was reduced by 50% at 2.5°C cultures relative to the −1.8 ◦C treatment, although there were no differences between both CO_2_ levels at 2.5°C [5]. Our results show a decrease of ~3% in PUFA content in the 750 μatm manipulation in relation to the 380 μatm CO_2_ treatment on both temperature levels, which is much smaller in relation to the above mentioned studies. These differences among the diatoms may be due to species-specific responses to CO_2_ within the same functional group [44]. For other algal taxa a similar response was observed in the Antarctic green algae *Pyramimonas gelidicola* by Wynn-Edwards et al. [45], who found a decrease of 34% in its PUFA content when cultured at elevated CO_2_ levels (993 μatm CO_2_), as well as in several other green algae in a study by Tsuzuki et al. [17] who reported a strong influence of high CO_2_ on the FA of *Chlorella vulgaris* cells and similar but smaller changes in *Chlamydomonas reinhardtii* and *Dunaliella tertiolecta*. Important to note is that the difference in the duration of high CO_2_ exposure in the studies cited above (short-term) compared to this study (long-term) may play a role in the observed differences in CO_2_ responses.

The low amount of PUFA observed in the 180 μatm CO_2_ treatment in relation to the 380 μatm CO_2_ cultures (difference of ~6% at 14°C and ~4% at 19°C) might be due to carbon allocation at low CO_2_ levels towards other essential processes, for instance cell division, which was kept relatively constant by *C*. *fusiformis* among the different treatments (S1C Fig). This kind of differentiate allocation of carbon between different biomolecules when cultured under low CO_2_ levels has been observed in the diatom *Chaetoceros wighamii* [23] and the green algae *Dunaliella salina* [46]. In the case of *C*. *fusiformis* the response to low CO_2_ seems to take place at the expense of physiologically important biomolecules such as PUFA and EA (Figs 1A and 3A). Further research is needed to determine how carbon is allocated among biomolecules and biological processes by *C*. *fusiformis* subjected to low CO_2_ levels. Although the mechanisms through which elevated CO_2_ affects algal FA are unclear, it has been proposed that an elevated SFA level in cells grown at high CO_2_ conditions is due to enhanced FA synthesis and accumulation [21]. It has been observed that environmentally high CO_2_ levels and related low pH can severely alter the cellular internal pH, thereby disturbing the tightly regulated homeostasis [22,47]. Consequently, elevated SFA synthesis under high CO_2_ conditions can be a mechanism to produce a less fluid cell membrane that is built of short-chained FA, making them less permeable, and helping in the regulation of the cellular homeostasis [7].

Temperature can affect the FA profile of algae [4,5,18]. Our long-term study showed a significant temperature-related effect on the FA composition of *C*. *fusiformis* in the 180 μatm CO_2_ treatment, with a higher amount of PUFA (and lower SFA) at 19°C in relation to the 14°C treatment (~ +3%). This observation agrees with results on the diatom *Chaetoceros muelleri* that exhibited a decrease in relative PUFA content when cultured at 35°C in contrast to 40°C [4]. However our results are contradictory to previous observations in which PUFA concentrations are generally inversely proportional to temperature [5,18]. The difference may be due to the length of the experiments and allocation of carbon within the cell [23,46] as mentioned above.

### Amino acids

Overall there is little information regarding the effects of CO_2_ on algal AA composition, and none within the range of future CO_2_ projections. The analysis of *C*. *fusiformis* showed a higher EA content at 380 μatm CO_2_ levels relative to the 750 μatm CO_2_ treatment (~ +6%) at 14°C. This is similar to observations of the green algae *Chlamydomonas reinhardtii* when comparing low (400 ppm CO_2_) to very high (5000 ppm CO_2_) CO_2_-enriched cultures, which showed that five out of six EA were significantly lower at the high CO_2_ treatments [25]. This EA change in *C*. *reinhardtii* was attributed to a down-regulation of the proteins related to the carbon concentrating mechanisms at high CO_2_ levels [25]. We would expect a similar mechanism underlying the EA shift observed in *C*. *fusiformis* as diatoms have been described to possess highly efficient carbon concentrating mechanisms, which are effectively down-regulated in some species by elevated CO_2_ concentrations [48]. It is noteworthy that there were lower EA amounts at the 180 μatm CO_2_ level than in the other CO_2_ treatments; this result, as in the case of PUFA, may be due to a differentiate carbon allocation towards other cellular biomolecules [23,46]. However, in this case it is more likely that the lower protein levels did not belong to the algal CCM complex in order to keep up a high uptake of inorganic carbon and constant growth in a process similar to what was observed in *Chlamydomonas reinhardtii* [25]. A more detailed study would be required to clarify the AA metabolic state of the algae under low CO_2_ conditions.

Our observations regarding the temperature effects on the relative AA composition of *C*. *fusiformis* showed that EA were more abundant at 19°C in relation to 14°C. This result agrees with findings in the green algae *Chlorella* strain MFD-1 which showed an EA increase from 31 to 36% when temperature was shifted from 15 to 25°C and *Nannochloropsis* strain MFD-2, in which EA also increased in content from 33 to 38% when the temperature was elevated from 15 to 35°C [24]. The results of both studies indicate that higher temperatures increase the EA content in algae.

The lack of CO_2_-related differences in relative EA content between the treatments at 19°C indicates that temperature has a greater influence on the algal EA composition than CO_2_, and can mask a CO_2_-related effect in the relative algal EA content below and above the 380 μatm CO_2_ levels. This could be due to temperature regulating a higher number of physiological processes in the cell than CO_2_. Consequently, the amount of synthesized protein and AA by temperature-influenced pathways might be higher than what is produced by CO_2_-influenced mechanisms. Nevertheless all this does not explain why proportionally more EA are produced at higher temperatures. A detailed study of the AA metabolism in these algae will be required to clarify this effect.

### Effect of the long-term conditioning to pCO_2_ and warming in *C*. *fusiformis*

In general the observed CO_2_ influence on the FA composition of *C*. *fusiformis* was comparatively smaller than observed in previous studies. A reason for this may be the experimental duration, with this experiment with >250 generations lasting much longer than previous studies [5,7,17], and hence allowing the possibility for *C*. *fusiformis* to undergo long-term acclimatization or adaptive evolution [49,50]. Recently Lohbeck et al. [51] showed that the CO_2_-sensitive coccolithophore *Emiliania huxleyi* was able to partly restore its calcification and growth rates through adaptive evolution when kept over 500 generations under high CO_2_ levels. Thus, CO_2_ and temperature related effects in the algal FA and EA compositions may also have been partly compensated after 250 generations in a process similar to the restored calcification in *E*. *huxleyi* observed by Lohbeck et al. [51]. However, the present data does not allow to distinguish if said process was due to acclimatization or evolutionary adaptation [49,52] in *C*. *fusifirmis*.

### Consequences of pCO_2_ and warming on the food quality of *C*. *fusiformis* in terms of fatty and amino acids

It has been shown that growth and reproduction of the copepod *Acartia tonsa* were constrained when fed with the diatom *Thalassiosira pseudonana* grown at 750 μatm CO_2_, which produced diatom cells with 20% less PUFA. The PUFA content decreased by 29% in the copepod, and caused a decrease in both somatic growth and egg production of ~85% [7]. Along the same lines, EA have a strong influence on aquatic grazers as observed in the copepods *Calanus finmarchicus* subjected to different diets [12]. It has been shown that the highest cumulative egg production typically correlates with the pool of EA in the food source, and that the high abundance of EA in the copepod diet promoted high fecundity and egg hatching success [12]. The present study is the first to report adverse effects of temperature and CO_2_ on the EA of a primary producer, and corroborate previous findings of such effects on essential PUFA content. Although the magnitude of these effects in the *C*. *fusiformis* FA is comparatively modest in relation to previous reports, possibly due to acclimatization or adaptation, their impacts can be amplified towards higher trophic levels. This can be observed in the results of Rossoll et al. [7] where the CO_2_ impact in the prey diatom PUFA was amplified 1.5 times in the copepod PUFA. Since about 20% of essential macromolecules like PUFA (and possibly EA) are incorporated into new biomass of organisms of the next trophic level [53], even small adverse effects of temperature and CO_2_ on the biochemistry of primary producers can be amplified in the marine food web [31,54].

Nonetheless, the effect of temperature and CO_2_ on the algal food quality for higher trophic levels will depend on the sensitivity of the different primary producers and on how ocean acidification affects the species composition of plankton assemblages, because different algal taxa possess contrasting biomolecular compositions [55–57] and sensitivities to CO_2_ [17,21,58].

The effect of ocean acidification and warming on the quality of diatoms as food source observed in the present work may have important effects in food webs as FA and AA produced by phytoplankton are incorporated into zooplankton and larval fish [7,59], which are as well an important food source for human populations [32]. Our results show that ocean acidification and warming can have unforeseen consequences in the physiology of primary producers which may cause indirect effects through trophic interactions, which need to be considered in future ocean scenarios.

## Supporting Information

S1 Fig Measured pH, calculated pCO_2_ and growth rate of the diatom *Cylindrotheca fusiformis* at the end of the experiment. The algae was cultured under three different CO_2_ conditions and two temperatures for >250 generations. a) The pH in the different treatments (n = 3). There is a significant difference in pH between the CO_2_ treatments (two-way ANOVA, F = 69.5, p<0.0001, df = 2), while there was no significant difference with the temperature treatments. b) Calculated pCO2 in the different treatments using pH and alkalinity measurements with the software CO2SYS (n = 3). There is a significant difference in pCO_2_ between the treatments (two-way ANOVA, F = 52.5, p<0.0001, df = 1). No significant difference was observed within the temperature treatments. c) Growth rate (n = 3). There is no significant difference between the CO_2_ treatments (two-way ANOVA, p>0.05), and although the diatoms showed a higher growth rate at 19°C, there was also no significant difference with the 14°C cultures (t-test, p>0.05). Error bars denote ± 1 standard deviation.
(TIFF)

S2 Fig Relative content of Eicosapentaenoic acid [EPA] and Leucine [Leu] in *Cylindrotheca fusiformis*. The diatom was cultured under three different CO_2_ conditions and two temperatures for >250 generations. a) The EPA showed significant differences in relation with CO_2_ (two-way ANOVA, F = 11.02, p = 0.001922, df = 2), while temperature and its interaction with CO_2_ were not significant (p>0.05). b) The Leu showed no significant differences between temperature or CO_2_ (p>0.05), however the interaction of temperature and CO_2_ was significant (two way ANOVA, F = 3.7, p = 0.057, df = 2). Error bars denote ± 1 standard deviation (n = 3, with exception of the 180 CO_2_-19°C treatment in (b) where n = 2).
(TIFF)

S3 Fig Load of the principal component analysis [PCA] of the fatty acids [FA] and amino acids [AA] of the diatom *Cylindrotheca fusiformis*. The algae was cultured under three different CO_2_ conditions and two temperatures for >250 generations. Only the FA and AA with a concentration above 1% were included in the analysis. a) Axis loads of the PCA analysis. The PUFA had a strong influence on the variance of both axis. ^#^ indicate PUFA. b) Axis loads of the PCA analysis. The EA had a strong influence on the variance of both axis. *indicate EA.
(TIFF)

S1 Table Relative content (%) of the Individual fatty acids [FA] measured in the diatom *Cylindrotheca fusiformis*. A total of 24 individual FA were identified and measured. The profile consisted of ~27% polyunsaturated (PUFA), ~ 23% monounsaturated (MUFA) and ~50% saturated (SFA) fatty acids.
(PDF)

S2 Table Relative content (%) of the Individual amino acids [AA] measured in the diatom *Cylindrotheca fusiformis*. A total of 12 AA were identified and quantified. The analysis of the specific AA groups showed that the Essential (EA) represented ~40% and Non-essential (NEA) the remaining ~60%.
(PDF)

S3 Table Result of the post-hoc statistical analysis of the fatty acids [FA] amino acids [AA] measured in the diatom *Cylindrotheca fusiformis*. The values given are p values. Values with p<0.05 are presented in bold. All treatment combinations are listed. PUFA, MUFA and SFA are poly, mono and saturated fatty acids, respectively. EA and NEA stand for essential and non-essential amino acids.
(PDF)

## References

[1] Rhein M, Rintoul SR, Aoki S, Campos E, Chambers D, Feely, RA et al. Observations: Ocean. In: Climate Change 2013: The Physical Science Basis. Contribution of Working Group I to the Fifth Assessment Report of the Intergovernmental Panel on Climate Change. In: Stocker TF, Qin D, Plattner G-K, Tignor M, Allen SK, et al., editors. Fifth Assessment Report of the Intergovernmental Panel on Climate Change. New York, USA. 2013; pp. 255–315.

[2] Doney SC, Fabry VJ, Feely RA, Kleypas JA. Ocean Acidification: The Other CO_2_ Problem. Ann Rev Mar Sci. 2009; 1: 169–192. 21141034 10.1146/annurev.marine.010908.163834

[3] Kroeker KJ, Kordas RL, Crim RN, Singh GG. Meta-analysis reveals negative yet variable effects of ocean acidification on marine organisms. Ecol Lett. 2010; 13: 1419–1434. 10.1111/j.1461-0248.2010.01518.x 20958904

[4] Rousch J, Bingham SE, Sommerfeld MR. Changes in fatty acid profiles of thermo-intolerant and thermo-tolerant marine diatoms during temperature stress. J Exp Mar Bio Ecol. 2003; 295: 145–156.

[5] Torstensson A, Hedblom M, Andersson J, Andersson MX, Wulff A. Synergism between elevated *p*CO_2_ and temperature on the Antarctic sea ice diatom *Nitzschia lecointei* . Biogeosciences. 2013; 10: 6391–6401.

[6] Winder M, Schindler D. Climate change uncouples trophic interactions in an aquatic ecosystem. Ecology. 2004; 85: 2100–2106.

[7] Rossoll D, Bermúdez R, Hauss H, Schulz KG, Riebesell U, Sommer U, et al Ocean acidification-induced food quality deterioration constrains trophic transfer. PLoS One. 2012; 7: e34737 10.1371/journal.pone.0034737 22509351 PMC3324536

[8] Winder M, Sommer U. Phytoplankton response to a changing climate. Hydrobiologia. 2012; 698: 5–16.

[9] Mouritsen O. Life-as a matter of fat 1st ed. Heidelberg: Springer;2005

[10] Young VR. Adult amino acid requirements: The case for a major revision in current recommendations. J Nutr. 1994; 124: 1517S – 1523S. 8064412 10.1093/jn/124.suppl_8.1517S

[11] Belitz D, Grosch W, Schieberle P. Amino acids, peptides and proteins. Food Chemistry. 2009;Vol. 34 pp. 8–33. 20351818

[12] Helland S, Nejstgaard J, Humlen R. Effects of season and maternal food on *Calanus finmarchicus* reproduction, with emphasis on free amino acids. Mar Biol; 2003 142: 1141–1151.

[13] Brett M, Müller-Navarra DC, Persson J. Crustacean zooplancton fatty acid composition In:. Arts M, Brett M, Kainz M, editors. Lipids in aquatic ecosystems. Dordrecht: Springer;2009

[14] Harrison P, Thompson P, Calderwood G. Effects of nutrient and light limitation on the biochemical composition of phytoplankton. J Appl Phycol. 1990; 2: 45–56.

[15] Reitan K, Rainuzzo J, Olsen Y. Effect of nutrient limitation on fatty acid and lipid content of marine microalgae. J Phycol. 1994; 30: 972–979.

[16] Roleda MY, Slocombe SP, Leakey RJG, Day JG, Bell EM, Stanley M. Effects of temperature and nutrient regimes on biomass and lipid production by six oleaginous microalgae in batch culture employing a two-phase cultivation strategy. Bioresour Technol. 2013; 129: 439–449. 10.1016/j.biortech.2012.11.043 23262022

[17] Tsuzuki M, Ohnuma E, Sato N, Takaku T, Kawaguchi A. Effects of CO_2_ concentration during growth on fatty acid composition in microalgae. Plant Physiol. 1990; 93: 851–856. 16667592 10.1104/pp.93.3.851 PMC1062600

[18] Van Wagenen J, Miller TW, Hobbs S, Hook P, Crowe B, Huesemann M. Effects of light and temperature on fatty acid production in *Nannochloropsis salina* . Energies. 2012; 5: 731–740.

[19] Riebesell U, Revill A, Holdsworth D, Volkman J. The effects of varying CO_2_ concentration on lipid composition and carbon isotope fractionation in *Emiliania huxleyi* . Geochim Cosmochim Acta. 2000; 64: 4179–4192.

[20] Fiorini S, Gattuso J-P, van Rijswijk P, Middelburg J. Coccolithophores lipid and carbon isotope composition and their variability related to changes in seawater carbonate chemistry. J Exp Mar Bio Ecol. 2010; 394: 74–85.

[21] Sato N, Tsuzuki M, Kawaguchi A. Glycerolipid synthesis in *Chlorella kessleri* 11 h II. Effect of the CO_2_ concentration during growth. Biochim Biophys Acta. 2003; 1633: 35–42. 12842193 10.1016/s1388-1981(03)00070-2

[22] Lane AE, Burris JE. Effects of Environmental pH on the Internal pH of *Chlorella pyrenoidosa*, *Scenedesmus quadricauda*, and *Euglena mutabilis* . Plant Physiol. 1981; 68: 439–442. 16661932 10.1104/pp.68.2.439 PMC427506

[23] De Castro Araújo S, Garcia VMT. Growth and biochemical composition of the diatom *Chaetoceros* cf. *wighamii* Brightwell under different temperature, salinity and carbon dioxide levels. I. Protein, carbohydrates and lipids. Aquaculture. 2005; 246: 405–412.

[24] James C, Al-Hinty S, Salman A. Growth and ω 3 fatty acid and amino acid composition of microalgae under different temperature regimes. Aquaculture. 1989; 77: 337–351.

[25] Renberg L, Johansson AI, Shutova T, Stenlund H, Aksmann A, Raven J, et al A metabolomic approach to study major metabolite changes during acclimation to limiting CO_2_ in *Chlamydomonas reinhardtii* . Plant Physiol. 2010; 154: 187–196. 10.1104/pp.110.157651 20634393 PMC2938146

[26] Müller-Navarra DC, Brett MT, Liston a M, Goldman CR. A highly unsaturated fatty acid predicts carbon transfer between primary producers and consumers. Nature. 2000; 403: 74–77. 10638754 10.1038/47469

[27] Müller-Navarra DC, Brett MT, Park S, Chandra S, Ballantyne AP, Zorita E, et al Unsaturated fatty acid content in seston and tropho-dynamic coupling in lakes. Nature. 2004; 427: 69–72. 14702086 10.1038/nature02210

[28] Jónasdóttir S, Visser A, Jespersen C. Assessing the role of food quality in the production and hatching of *Temora longicornis* eggs. Mar Ecol Prog Ser. 2009; 382: 139–150.

[29] Fink P, Pflitsch C, Marin K. Dietary essential amino acids affect the reproduction of the keystone herbivore *Daphnia pulex* . PLoS One. 2011; 6: e28498 10.1371/journal.pone.0028498 22163027 PMC3230618

[30] St. John M. Diatom production in the marine environment: implications for larval fish growth and condition. ICES J Mar Sci. 2001; 58: 1106–1113.

[31] Izquierdo M., Fernández-Palacios H, Tacon AG. Effect of broodstock nutrition on reproductive performance of fish. Aquaculture. 2001; 197: 25–42.

[32] Food and Agriculture Organization of the United Nations. The State of World Fisheries and Aquaculture 2010. Séligny J de, Grainger R, editors. Rome; 2010.

[33] Tatters A, Roleda MY, Schnetzer A, Fu F, Hurd C, Boyd PW, et al Short-and long-term conditioning of a temperate marine diatom community to acidification and warming. Phil Trans R Soc Lond B. 2013;368: 20120437 10.1098/rstb.2012.0437 23980240 PMC3758171

[34] Currie KI, Reid MR, Hunter KA. Interannual variability of carbon dioxide drawdown by subantarctic surface water near New Zealand. Biogeochemistry. 2011; 104: 23–34.

[35] Collins S, Bell G. Evolution of natural algal populations at elevated CO_2_ . Ecol Lett.2006; 9: 129–135. 16958877 10.1111/j.1461-0248.2005.00854.x

[36] Hare C, Leblanc K, DiTullio G, Kudela R, Zhang Y, Lee PA, et al Consequences of increased temperature and CO_2_ for phytoplankton community structure in the Bering Sea. Mar Ecol Prog Ser. 2007; 352: 9–16.

[37] Guillard R, Ryther J. Studies of marine planktonic diatoms: I. *Cyclotella nana* Hustedt, and *Detonula confervacea* (Cleve) gran. Can J Microbiol. 1962; 8: 229–239. 13902807 10.1139/m62-029

[38] McGraw CM, Cornwall CE, Reid MR, Currie KI, Hepburn CD, Boyd P, et al An automated pH-controlled culture system for laboratory-based ocean acidification experiments. Limnol Oceanogr Methods. 2010; 8: 686–694.

[39] Dickson A,G, Afghan JD, Anderson GC. Reference materials for oceanic CO_2_ analysis: a method for the certification of total alkalinity. Mar Chem. 2003; 80: 185–197.

[40] Lewis E, Wallace D, Allison L. Program developed for CO_2_ system calculations. ORNL/CDIAC-105. Carbon Dioxide Information Analysis Center, Oak Ridge National Laboratory, U.S. Department of Energy.1998. Oak Ridge, Tennessee, USA.

[41] Breteler W, Schogt N, Baas M. Trophic upgrading of food quality by protozoans enhancing copepod growth: role of essential lipids. Mar Biol. 1999; 135: 191–198.

[42] Badawy AA-B, Morgan CJ, Turner JA. Application of the Phenomenex EZ:faast amino acid analysis kit for rapid gas-chromatographic determination of concentrations of plasma tryptophan and its brain uptake competitors. Amino Acids. 2008; 34: 587–596. 18071842 10.1007/s00726-007-0012-7 PMC2797848

[43] R Development Core Team. R: A language and environment for statistical computing. Vienna. 2015. Available: http://www.r-project.org/.

[44] Fabry VJ. Marine calcifiers in a high-CO_2_ ocean. Science. 2008; 320: 1020–1022. 10.1126/science.1157130 18420898

[45] Wynn-Edwards C, King R, Davidson A, Wright S, Nichols P, Wotherspoon S, et al Species-specific variations in the nutritional quality of southern ocean phytoplankton in response to elevated pCO_2_ . Water. 2014; 6: 1840–1859.

[46] Giordano M, Bowes G. Gas exchange and C allocation in *Dunaliella salina* cells in response to the N source and CO_2_ concentration used for growth. Plant Physiol. 1997: 1049–1056. 12223857 10.1104/pp.115.3.1049 PMC158568

[47] Suffrian K, Schulz KG, Gutowska MA, Riebesell U, Bleich M. Cellular pH measurements in *Emiliania huxleyi* reveal pronounced membrane proton permeability. New Phytol. 2011; 190: 595–608. 10.1111/j.1469-8137.2010.03633.x 21294736

[48] Burkhardt S, Amoroso G. CO_2_ and HCO_3_ ^-^uptake in marine diatoms acclimated to different CO_2_ concentrations. Limnol Oceanogr. 2001; 46: 1378–1391.

[49] Somero GN. The physiology of climate change: how potentials for acclimatization and genetic adaptation will determine “winners” and “losers”. J Exp Biol. 2010; 213: 912–920. 10.1242/jeb.037473 20190116

[50] Reusch TBH. Climate change in the oceans: evolutionary versus phenotypically plastic responses of marine animals and plants. Evol Appl. 2013; 7: 104–122. 10.1111/eva.12109 24454551 PMC3894901

[51] Lohbeck KT, Riebesell U, Reusch TBH. Adaptive evolution of a key phytoplankton species to ocean acidification. Nat Geosci. 2012; 5: 346–351.

[52] Reusch TBH, Boyd PW. Experimental evolution meets marine phytoplankton. Evolution. 2013; 67: 1849–1859. 10.1111/evo.12035 23815643

[53] Gladyshev MI, Sushchik NN, Anishchenko O V, Makhutova ON, Kolmakov VI, Kalachova GS, et al Efficiency of transfer of essential polyunsaturated fatty acids versus organic carbon from producers to consumers in a eutrophic reservoir. Oecologia. 2011; 165: 521–531. 10.1007/s00442-010-1843-6 21107868

[54] Fraser A, Sargent J, Gamble J. Lipid class and fatty acid composition of *Calanus finmarchicus* (Gunnerus), *Pseudocalanus sp*. and *Temora longicornis* Muller from a nutrient-enriched seawater enclosure. J Exp Mar Biol.1989; 130: 81–92.

[55] Dunstan GA, Volkman JK, Jeffrey SW, Barrett SM. Biochemical composition of microalgae from the green algal classes Chlorophyceae and Prasinophyceae. 2. Lipid classes and fatty acids. J Exp Mar Bio Ecol. 1992; 161: 115–134.

[56] Viso A, Marty J. Fatty acids from 28 marine microalgae. Phytochemistry. 1993; 34: 1521–1533.

[57] Zhukova N, Aizdaicher N. Fatty acid composition of 15 species of marine microalgae. Phytochemistry. 1995; 39: 351–356.

[58] Sato N. Modulation of lipid and fatty acid content by carbon dioxide in *Chlamydomonas reinhardtii* . Plant Sci.1989; 61: 17–21.

[59] Fraser AJ, Sargent JR, Gamble JC, Seaton DD. Formation and transfer of fatty acids in an enclosed marine food chain comprising phytoplankton, zooplankton and herring (*Clupea harengus* L.) larvae. Mar Chem. 1989; 27: 1–18.

