# Supplementary figures and images for: Long-Term Conditioning to Elevated pCO2 and Warming Influences the Fatty and Amino Acid Composition of the Diatom Cylindrotheca fusiformis
Source: PLoS One. 2015 May 13;10(5):e0123945. doi: 10.1371/journal.pone.0123945 (PMC4430207; doi:10.1371/journal.pone.0123945)

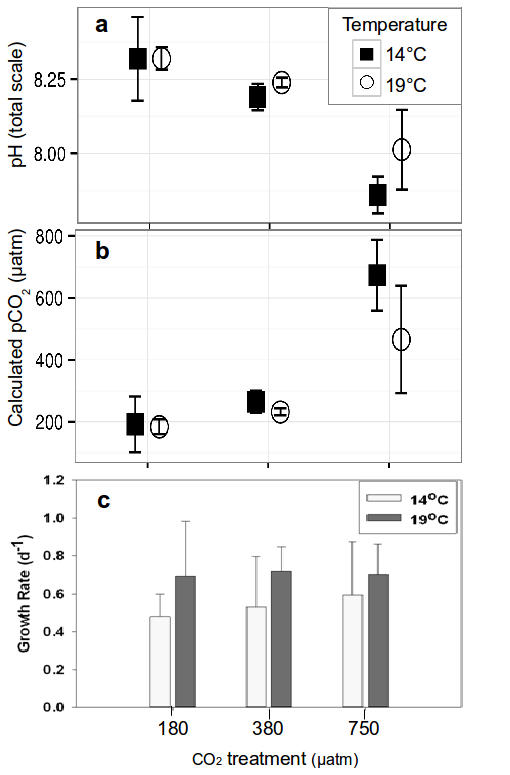

Supplement: S1 Fig — The algae was cultured under three different CO2 conditions and two temperatures for >250 generations. a) The pH in the different treatments (n = 3). There is a significant difference in pH between the CO2 treatments (two-way ANOVA, F = 69.5, p<0.0001, df = 2), while there was no significant difference with the temperature treatments. b) Calculated pCO2 in the different treatments using pH and alkalinity measurements with the software CO2SYS (n = 3). There is a significant difference in pCO2 between the treatments (two-way ANOVA, F = 52.5, p<0.0001, df = 1). No significant difference was observed within the temperature treatments. c) Growth rate (n = 3). There is no significant difference between the CO2 treatments (two-way ANOVA, p>0.05), and although the diatoms showed a higher growth rate at 19°C, there was also no significant difference with the 14°C cultures (t-test, p>0.05). Error bars denote ± 1 standard deviation. (TIFF) [file pone.0123945.s001.tiff]

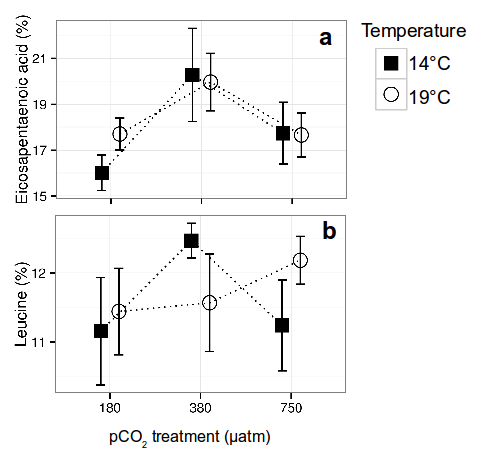

Supplement: S2 Fig — The diatom was cultured under three different CO2 conditions and two temperatures for >250 generations. a) The EPA showed significant differences in relation with CO2 (two-way ANOVA, F = 11.02, p = 0.001922, df = 2), while temperature and its interaction with CO2 were not significant (p>0.05). b) The Leu showed no significant differences between temperature or CO2 (p>0.05), however the interaction of temperature and CO2 was significant (two way ANOVA, F = 3.7, p = 0.057, df = 2). Error bars denote ± 1 standard deviation (n = 3, with exception of the 180 CO2-19°C treatment in (b) where n = 2). (TIFF) [file pone.0123945.s002.tiff]

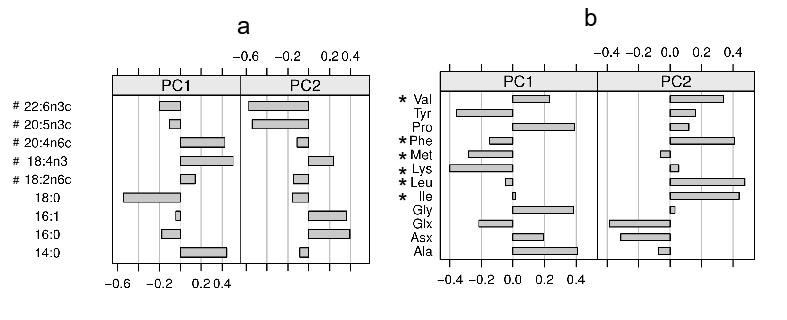

Supplement: S3 Fig — The algae was cultured under three different CO2 conditions and two temperatures for >250 generations. Only the FA and AA with a concentration above 1% were included in the analysis. a) Axis loads of the PCA analysis. The PUFA had a strong influence on the variance of both axis. # indicate PUFA. b) Axis loads of the PCA analysis. The EA had a strong influence on the variance of both axis. *indicate EA. (TIFF) [file pone.0123945.s003.tiff]
