# Supplementary material for: Long-Term Conditioning to Elevated pCO2 and Warming Influences the Fatty and Amino Acid Composition of the Diatom Cylindrotheca fusiformis
Source: PLoS One. 2015 May 13;10(5):e0123945. doi: 10.1371/journal.pone.0123945 (PMC4430207; doi:10.1371/journal.pone.0123945)
Supplement: S1 Table — A total of 24 individual FA were identified and measured. The profile consisted of ~27% polyunsaturated (PUFA), ~ 23% monounsaturated (MUFA) and ~50% saturated (SFA) fatty acids. (PDF) [file pone.0123945.s004.pdf]

Supplemental Table.

Table S1: Relative content (%) of the Individual fatty acids [FA] measured in the diatom *Cylindrotheca fusiformis*. A total of 24 individual FA were identified and measured. The profile consisted of ~27% polyunsaturated (PUFA), ~ 23% monounsaturated (MUFA) and ~50% saturated (SFA) fatty acids.

	Temp. °C	14	14	14	14	14	14	14	14	14	19	19	19	19	19	19	19	19	19
	CO_2 (µatm)	180	180	180	380	380	380	750	750	750	180	180	180	380	380	380	750	750	750
Type																			
PUFA	18:2n6c	0.9	0.7	0.7	0.9	0.7	0.8	0.9	0.7	0.8	1.0	1.1	1.0	1.2	1.3	1.1	1.0	0.9	1.0
PUFA	18:2n6t	0.1	0.1	0.1	0.0	0.0	0.0	0.1	0.1	0.0	0.1	0.0	0.0	0.0	0.0	0.0	0.0	0.0	0.0
PUFA	18:3n4	0.1	0.0	0.0	0.0	0.0	0.0	0.0	0.0	0.0	0.1	0.0	0.1	0.1	0.1	0.0	0.0	0.0	0.0
PUFA	18:3n6	0.0	0.0	0.0	0.0	0.0	0.0	0.1	0.0	0.0	0.0	0.0	0.0	0.0	0.0	0.0	0.0	0.0	0.0
PUFA	18:4n3	3.8	2.9	2.4	3.7	3.2	3.9	3.7	3.1	3.5	3.4	4.0	3.8	3.4	3.5	3.0	3.9	3.9	4.0
PUFA	20:3n6	0.1	0.2	0.1	0.2	0.1	0.1	0.1	0.2	0.2	0.1	0.1	0.1	0.1	0.1	0.0	0.2	0.1	0.2
PUFA	20:4n6c	2.1	2.0	1.8	2.2	2.1	2.3	2.0	2.3	2.2	2.7	2.6	2.7	2.7	2.8	3.5	3.7	3.4	3.4
PUFA	20:5n3c	16.5	15.1	16.4	18.0	21.2	21.7	16.3	17.9	19.0	17.7	17.0	18.4	19.4	19.1	21.4	17.2	18.8	17.0
PUFA	22:2n6c	0.1	0.1	0.1	0.1	0.1	0.0	0.0	0.1	0.1	0.2	0.1	0.0	0.1	0.0	0.0	0.1	0.2	0.1
PUFA	22:6n3c	2.5	2.5	2.8	3.2	3.9	4.1	2.9	3.3	3.2	2.7	2.7	2.9	3.6	4.0	4.4	2.9	2.9	2.8
MUFA	14:1	0.1	0.0	0.3	0.0	0.1	0.0	0.1	0.1	0.0	0.1	0.1	0.1	0.1	0.1	0.2	0.1	0.0	0.1
MUFA	16:1	21.3	17.7	17.3	19.6	21.7	20.3	18.6	21.6	21.3	19.2	19.7	19.9	23.3	22.2	21.8	26.2	26.7	26.3
MUFA	18:1n9c	0.6	0.8	0.6	0.4	0.5	0.6	0.6	0.8	0.6	0.4	0.6	0.5	0.5	0.6	0.4	0.5	0.5	0.5
MUFA	18:1n9t	0.0	0.0	0.0	0.0	0.0	0.0	0.0	0.0	0.0	0.1	0.1	0.0	0.0	0.0	0.0	0.0	0.0	0.0
MUFA	20:1n9c	0.0	0.0	0.0	0.0	0.0	0.0	0.0	0.0	0.0	0.1	0.0	0.0	0.0	0.0	0.0	0.0	0.0	0.0
MUFA	22:1n9c	0.1	0.0	0.3	0.0	0.2	0.1	0.3	0.2	0.0	0.1	0.0	0.0	0.0	0.0	0.0	0.0	0.0	0.0
MUFA	24:1n9c	0.1	0.0	0.2	0.1	0.2	0.2	0.1	0.2	0.1	0.1	0.1	0.0	0.1	0.1	0.1	0.1	0.1	0.1
SFA	10:0	0.0	0.0	0.0	0.0	0.0	0.0	0.0	0.0	0.0	0.0	0.0	0.0	0.0	0.0	0.0	0.0	0.0	0.0
SFA	12:0	0.1	0.1	0.1	0.1	0.1	0.1	0.1	0.1	0.1	0.1	0.1	0.0	0.1	0.1	0.1	0.0	0.0	0.0
SFA	14:0	22.2	23.5	19.1	24.9	20.6	19.4	24.9	20.4	21.3	19.6	20.9	19.5	20.1	19.0	19.0	19.5	19.1	19.5
SFA	16:0	25.2	25.8	25.4	20.9	18.0	18.3	22.7	21.2	20.4	23.9	24.8	23.1	18.9	20.2	17.7	18.0	17.3	19.1
SFA	18:0	2.8	6.8	10.3	4.9	5.8	6.2	5.4	6.5	5.2	5.2	3.4	4.3	3.7	4.8	4.3	3.5	3.3	3.6
SFA	20:0	0.1	0.1	0.2	0.1	0.1	0.1	0.1	0.1	0.1	0.1	0.1	0.1	0.1	0.1	0.0	0.0	0.1	0.0
SFA	24:0	0.1	0.2	0.3	0.1	0.1	0.2	0.1	0.2	0.2	0.1	0.2	0.2	0.2	0.2	0.2	0.1	0.1	0.1
