# Supplementary material for: Long-Term Conditioning to Elevated pCO2 and Warming Influences the Fatty and Amino Acid Composition of the Diatom Cylindrotheca fusiformis
Source: PLoS One. 2015 May 13;10(5):e0123945. doi: 10.1371/journal.pone.0123945 (PMC4430207; doi:10.1371/journal.pone.0123945)
Supplement: S2 Table — A total of 12 AA were identified and quantified. The analysis of the specific AA groups showed that the Essential (EA) represented ~40% and Non-essential (NEA) the remaining ~60%. (PDF) [file pone.0123945.s005.pdf]

Supplemental Table.

Table S2: Relative content (%) of the Individual amino acids [AA] measured in the diatom *Cylindrotheca fusiformis.* A total of 12 AA were identified and quantified. The analysis of the specific AA groups showed that the Essential (EA) represented ~40% and Non-essential (NEA) the remaining ~60%.

	Temp. °C	14	14	14	14	14	14	14	14	14	19	19	19	19	19	19	19	19
	CO_2 (µatm)	180	180	180	380	380	380	750	750	750	180	180	380	380	380	750	750	750
**Type**																		
**E**	Val	7.57	8.00	5.13	7.80	8.02	7.72	7.08	7.69	5.58	6.70	6.07	7.06	7.49	6.70	6.66	7.67	6.85
**E**	Ile	5.28	5.27	3.28	6.83	5.33	6.02	5.14	5.32	3.81	5.12	4.97	5.38	4.91	4.98	4.81	6.09	5.65
**E**	Leu	10.75	12.06	10.67	12.75	12.26	12.39	10.57	11.27	11.88	11.00	11.89	11.60	12.25	10.85	12.29	11.80	12.46
**E**	Lys	2.90	2.70	4.98	6.21	4.16	3.87	5.64	2.62	4.77	6.38	8.09	6.80	4.37	5.86	7.55	4.94	7.46
**E**	Met	1.90	1.23	2.56	2.02	1.95	1.38	1.43	1.01	1.61	2.06	2.54	2.16	2.51	2.44	2.31	2.86	2.21
**E**	Phe	5.27	6.55	5.34	7.66	5.76	6.56	5.71	5.39	6.87	5.37	6.71	6.32	6.06	5.86	6.42	5.94	6.77
**NE**	Pro	7.10	6.61	6.04	6.70	6.62	6.61	6.70	6.35	6.38	5.82	5.32	5.83	5.64	5.18	6.42	6.05	5.20
**NE**	Tyr	1.59	2.24	2.30	2.83	1.52	2.67	2.14	1.71	2.87	2.53	3.05	2.28	2.00	2.68	2.99	2.71	3.25
**NE**	Ala	16.03	14.16	14.14	12.81	14.83	13.52	12.82	14.75	15.43	12.87	11.50	14.35	13.45	12.19	13.31	13.71	11.04
**NE**	Asx	13.75	13.57	14.34	12.70	14.09	12.75	15.12	13.18	14.97	13.02	12.61	11.67	13.45	13.62	12.79	12.89	12.08
**NE**	Glx	12.33	12.41	18.01	9.99	11.17	13.26	16.03	16.36	10.59	16.65	16.45	12.48	14.51	18.84	10.02	11.36	15.98
**NE**	Gly	15.54	15.21	13.21	11.71	14.27	13.25	11.62	14.35	15.24	12.48	10.81	14.08	13.35	10.82	14.43	14.00	11.04
