# Supplementary material for: Long-Term Conditioning to Elevated pCO2 and Warming Influences the Fatty and Amino Acid Composition of the Diatom Cylindrotheca fusiformis
Source: PLoS One. 2015 May 13;10(5):e0123945. doi: 10.1371/journal.pone.0123945 (PMC4430207; doi:10.1371/journal.pone.0123945)
Supplement: S3 Table — The values given are p values. Values with p<0.05 are presented in bold. All treatment combinations are listed. PUFA, MUFA and SFA are poly, mono and saturated fatty acids, respectively. EA and NEA stand for essential and non-essential amino acids. (PDF) [file pone.0123945.s006.pdf]

Supplemental Table.

Table S3: Result of the post-hoc statistical analysis of the fatty acids [FA] amino acids [AA] measured in the diatom *Cylindrotheca fusiformis.* The values given are p values. Values with p<0.05 are presented in bold. All treatment combinations are listed. PUFA, MUFA and SFA are poly, mono and saturated fatty acids, respectively. EA and NEA stand for essential and non-essential amino acids.

	Fatty Acids			Amino Acids	
**Treatment Combination (pCO_2:Temperature)**	**PUFA**	**MUFA**	**SFA**	**EA**	**NEA**
**380:14-180:14**	**0.003**	0.999	**0.002**	**0.039**	**0.039**
**750:14-180:14**	0.250	0.999	0.085	0.998	0.998
**180:19-180:14**	**0.016**	1.000	0.113	0.187	0.187
**380:19-180:14**	**0.001**	0.759	**0.004**	0.202	0.202
**750:19-180:14**	**0.025**	0.849	0.122	**0.020**	**0.020**
**750:14-380:14**	0.132	0.975	0.299	0.075	0.075
**180:19-380:14**	0.289	1.000	0.234	0.987	0.987
**380:19-380:14**	0.980	0.913	0.998	0.891	0.891
**750:19-380:14**	0.763	0.961	0.219	0.998	0.998
**180:19-750:14**	0.994	0.989	1.000	0.310	0.310
**380:19-750:14**	**0.043**	0.547	0.492	0.351	0.351
**750:19-750:14**	0.709	0.653	1.000	**0.038**	**0.038**
**380:19-180:19**	0.102	0.866	0.401	0.999	0.999
**750:19-180:19**	0.938	0.931	1.000	0.909	0.909
**750:19-380:19**	0.385	1.000	0.378	0.687	0.687
